# COVID-19 infection among healthcare workers: a cross-sectional study in southwest Iran

**DOI:** 10.1186/s12985-021-01532-0

**Published:** 2021-03-17

**Authors:** Golnar Sabetian, Mohsen Moghadami, Leila Hashemizadeh Fard Haghighi, Reza Shahriarirad, Mohammad Javad Fallahi, Naeimehossadat Asmarian, Yalda Sadat Moeini

**Affiliations:** 1 Trauma Research Center, Shiraz University of Medical Sciences, Shiraz, Iran; 2 Clinical Microbiology Research Center, Shiraz University of Medical Sciences, Shiraz, Iran; 3 Vice Chancellor for Health Affairs Center of Disease Control (CDC), Shiraz University of Medical Sciences, Shiraz, Iran; 4 Student Research Committee, Shiraz University of Medical Sciences, Shiraz, Iran; 5 Thoracic and Vascular Surgery Research Center, Shiraz University of Medical Sciences, Shiraz, Iran; 6 Department of Internal Medicine, Nemazee Hospital, Shiraz University of Medical Sciences, Shiraz, Iran; 7 Anesthesiology and Critical Care Research Center, Shiraz University of Medical Sciences, Shiraz, Iran

**Keywords:** Healthcare workers, COVID-19, Iran

## Abstract

**Objective:**

With the novel coronavirus pandemic, the impact on the healthcare system and workers cannot be overlooked. However, studies on the infection status of medical personnel are still lacking. It is imperative to ensure the safety of health-care workers (HCWs) not only to safeguard continuous patient care but also to ensure they do not transmit the virus, therefore evaluation of infection rates in these groups are indicated.

**Methods:**

Demographic and clinical data regarding infected cases among HCWs of Fars, Iran with positive SARS‐CoV‐2 PCR tests were obtained from 10th March to 17th May 2020.

**Results:**

Our data demonstrated a rate of 5.62% (273 out of 4854 cases) infection among HCW, with a mean age of 35 years and a dominance of female cases (146 cases: 53.5%). The majority of infected cases were among nurses (51.3%), while the most case infection rate (CIR) was among physicians (27 positive cases out of 842 performed test (3.2%)). Also, the highest rate of infection was in the emergency rooms (30.6%). Also, 35.5% of the patients were asymptomatic and the most frequent clinical features among symptomatic patients were myalgia (46%) and cough (45.5%). Although 5.5% were admitted to hospitals, there were no reports of ICU admission. Furthermore, 10.3% of the cases reported transmitting the infection to family and friends. Regarding safety precautions, 1.6% didn't wear masks and 18.7% didn't use gloves in work environments.

**Conclusion:**

HCWs are among the highest groups at risk of infection during the COVID-19 pandemic; therefore, evaluating infection rates and associated features is necessary to improve and adjust protective measures of these vulnerable, yet highly essential group.

**Supplementary Information:**

The online version contains supplementary material available at 10.1186/s12985-021-01532-0.

## Background

In late December 2019, China reported an outbreak of viral pneumonia in Wuhan, Hubei Province, China, which spread rapidly to other areas [1, 2]. The novel coronavirus disease 2019 (Also known as COVID-19) caused by the severe acute respiratory syndrome coronavirus 2 (SARS-CoV-2) is a global concern and has become a significant health problem since the number of infected cases and affected countries has escalated rapidly [3]. On March 11, 2020, the World Health Organization (WHO) confirmed COVID-19 a pandemic and as of December 8th, over sixty-eight million cases of COVID have been reported with a death toll of over one and a half million patients and only around 47 million recovered cases in 218 countries and territories worldwide.

Amongst the highest population at the risk of exposure to the disease are the health-care workers (HCWs). Previous experiences of a similar disease, the severe acute respiratory syndrome (SARS), have left behind a distressing toll on the health-care providers. During the SARS outbreak in 2002, WHO confirmed 8098 cases and 774 (9.6%) deaths of which HCW accounted for 1707 (21%) cases. Also, Singapore reported 41% of 238 probable SARS cases in Singapore occurred in health-care workers [4]. With the ongoing pandemic of the COVID-19, occupational contact of HCWs is therefore among the most vital concerns which need to be addressed comprehensively and decisively. It is imperative to ensure the safety of HCWs not only to safeguard continuous patient care but also to ensure they do not transmit the virus. Reports have said that until 5th June 2020, at least 90,000 healthcare workers have been infected by COVID-19 and more than 260 nurses have lost their lives to the pandemic [5].

Iran was one of the top ten and hardest-hit countries by the COVID-19 pandemic. The outbreak of coronavirus in Iran was officially confirmed in Qom on February 19, 2020 [6], however, until 8th December, over one million cases have been reported with a death toll of above 50,000 cases. Here we report the incidence of COVID-19 infection among HCWs in Fars province, as one of the most substantial foci of the disease in southern Iran.

## Materials and methods

### Data collection

The prevention and control center of the 2019 novel Coronavirus Disease (COVID-19) was launched on February 20th, 2020, to detect the spread of the COVID-19 in Fars Province, Iran’s fourth most populated province. In this retrospective cross-sectional study, data regarding infected cases among HCWs and hospital staff working in Fars province were obtained from 10th March to 17th May 2020. The study population consisted of all health-care personnel working in either public or private hospitals in Fars province. These data were recorded from 44 private and community hospitals throughout the province. All personal underwent screening process at least once, while further tests were administered in personnel who developed COVID-19 related symptoms. Furthermore, personnel with shifts in COVID-19 specific wards underwent monthly PCR for SARS-CoV-2. Individuals with a history of close contact with a suspicious case outside of the workplace such as family members or close friends were excluded from our study. Infection with COVID-19 was confirmed by Real-time reverse transcriptase-polymerase chain reaction (RT-PCR). Testing was done for individuals based on the presentation of symptoms or in cases with unprotected close contact with confirmed COVID-19 case. RT-PCR assays were performed according to the protocol established by the WHO and previous studies [7–9]. The patient’s demographic information, signs and symptoms, radiological findings, occupation, and working location, along with safety protocols that they used and reports of infection among family and friends were recorded by contacting the patient and filling out a pre-designed form (Additional file 1). Also, signs and symptoms after recovery were documented. Furthermore, data regarding the total number of HCWs and also the total number of infected cases in the mentioned timeline in the studied centers along with features such as age, sex, and occupation were recorded for comparison.

### Statistical analysis

Statistical differences were assessed by Pearson's chi-square for categorical variables and Student's t test was used to compare means of quantitative data. All analyses were performed in SPSS version 26.0 while a P < 0.05 was considered statistically significant. Measurement data were described by mean ± standard deviation (SD) and enumeration data were described by number (%).

## Results

**Fig. 1:**
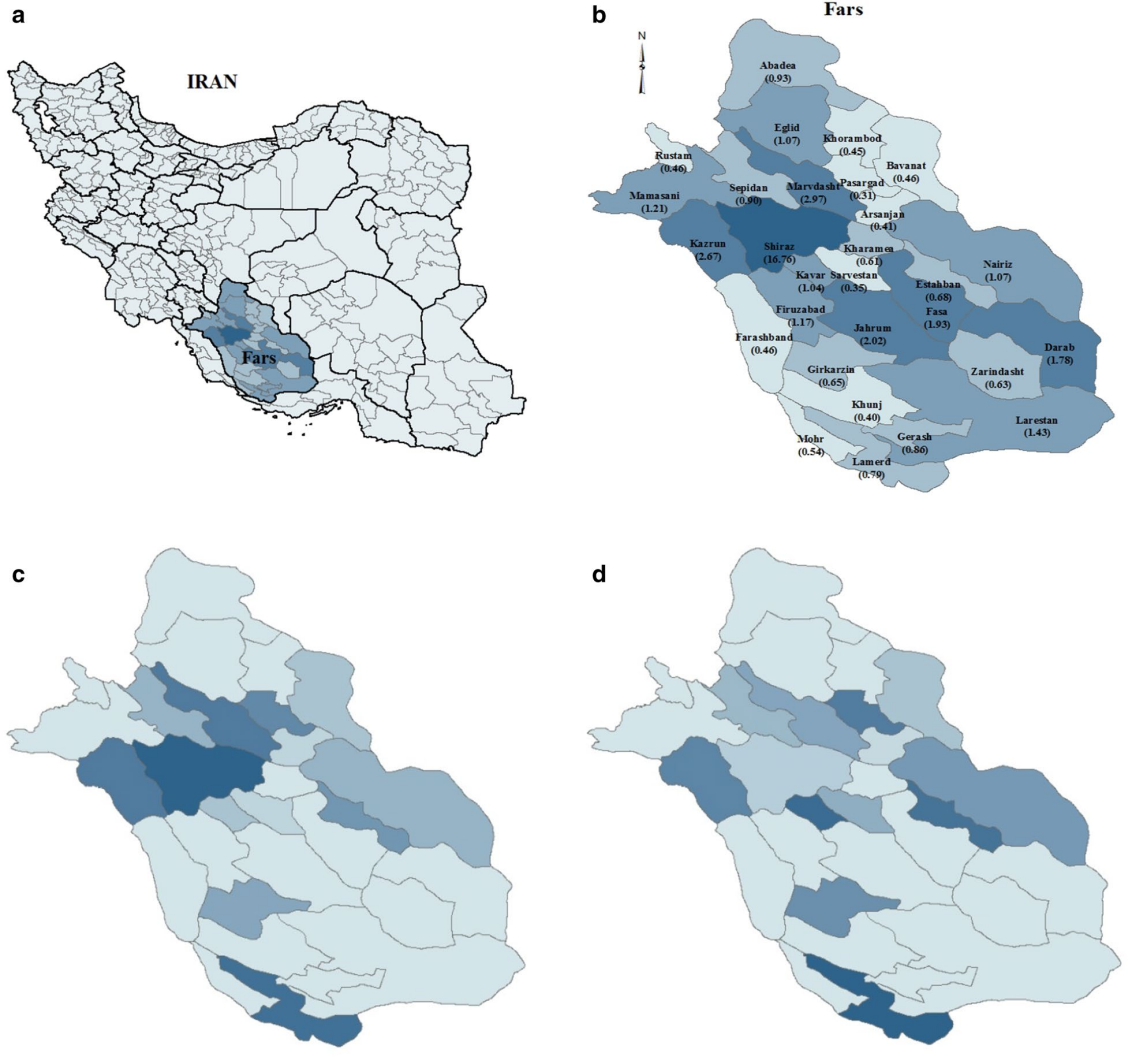
Geographic information system mapping of the novel coronavirus disease 2019 (COVID-19) infection in Fars province. **a** Geographical location of Fars province based on the map of Iran **b** population of cities in Fars province (× 10^5^) **c** COVID-19 infection rate among healthcare workers (HCW) based on number of cases among cities in Fars province; **d** COVID-19 infection ratio based on total healthcare workers among cities in Fars province (percentage of number of HCW with positive COVID-19 divided by total number of HCW in the mentioned city)

A total of 4854 cases of positive PCR COVID-19 individuals were documented, with a mean age of 40.77 (SD = 17.99, range 23—66) years with a majority of males (27.7%). The highest frequency of infected cases was among the age group of 25–45 years old. There was also a significant correlation among the age groups and also the gender of the patients. Our results showed that 280 HCWs were infected with COVID-19, in which after evaluation of the previous history of infection among family and close friends, seven were excluded. Therefore, this study was based on 273 (5.62% of total cases) cases of HCW cases that were infected based on an occupational source. Figure 1 demonstrates the geographic information system mapping of our study.

Among the selected patients, the age ranged from 23 to 66 years (mean = 35.02; SD = 8.34) while their work experience ranging from 3 months to 29 years (mean = 9.28; SD = 6.82). Table 1 demonstrates the demographic features of COVID-19 infected patients among the general population and hospital staff employees. As demonstrated, there was a significant difference among the age group and also the gender of infected patients among the two populations, with higher infection rates of females in HCWs compared to males in the general population. Also, the age group of 25–45 years showed significantly higher rates of infection in HCWs compared to the general population, while for other age groups, the general population had higher infection rates compared to HCWs.

**Table 1:** Demographic features of COVID-19 infected patients among the general population and hospital staff employees in Fars province, southern Iran

Variable	General population *n* = 4854		Hospital staff *n* = 273		P-value between populations*
	Frequency (%)	P value*	Frequency (%)	P value*	
*Age group*					
< 25	609 (16)	< 0.001	15 (5.5)	< 0.001	< 0.001
25 – 45	1863 (49)		229 (84.5)		
> 45	1327 (34.9)		27 (10)		
*Gender*					
Female	2039 (43.6)	< 0.001	146 (53.5)	0.250	0.001
Male	2639 (56.4)		127 (46.5)		

* Chi-square test, significance level set at 0.05

Among the patients in our study, the majority of infected cases were among nurses (51.3%) and were from personnel of emergency wards (30.6%).

Data regarding the number of performed tests among medical staff and HCWs were obtained. Among 10,707 total performed tests among hospital staff, 273 positive cases were detected (2.5%). These tests were among 44 private and public hospitals and centers in Fars province, while most of the reports were from Shiraz, the capital of the province (Additional file 2).

Case infection rate (CIR) in our study is defined as the number of infections among the total number of tests performed in the index group; which included 138 out of 6183 (2.23%) positive cases in nurses; 27 out of 842 (3.2%) in physicians; in paraclinical fields 27 out of 1165 (2.32%), among 22 out of 2517 (0.87%). Table 2 demonstrates the occupational features of HCWs.

**Table 2:** Occupational features of health care workers infected with the novel coronavirus 2019 in Fars, Iran

Variable	Frequency	Percentage (%)	P value*
*Years of experience*			
< 10	78	50.6	< 0.001
10–20	68	44.2	
> 20	8	5.2	
*Occupation*			
Nurse	138	51.3	< 0.001
Security and others	30	11.2	
Hospital staff	25	9.3	
Office workers	22	8.2	
Specialist	12	4.5	
Laboratory staff	10	3.7	
Midwifery	8	3	
General physician	8	3	
Radiology technician	8	3	
Medical student	4	1.5	
Fellowship	3	1.1	
Physiotherapist	1	0.4	
*Residence*			
Shiraz, capital of Fars province	151	58.3	0.008
Others cities in the province	108	41.7	
*Work location*			
Emergency wards	60	30.6	< 0.001
Wards (infectious and non-infectious)	39	19.9	
Hospital service	39	19.3	
Operating room	35	17.9	
Laboratory	9	4.6	
Radiology department	7	3.6	
Admission desk	5	2.6	
Security	4	2	

*Chi-square test, significance level set at 0.05

Based on our results, 220 (80.6%) of our patients had no underlying disease, while 48 (17.6%) had one, three (1.1%) had two, and two (0.7%) had three underlying diseases. The clinical features are demonstrated in Table 3. As shown, 97 (35.5%) of patients were asymptomatic.

**Table 3:** Clinical features and underlying diseases of health care workers infected with the novel coronavirus 2019

Variable	Frequency	Percentage (%)
*Underlying disease*		
Cerebrovascular disease	8	2.9
Immunodeficiency	7	2.6
Respiratory disease	7	2.6
Corticosteroid use	7	2.6
Gastrointestinal disease	5	1.8
Others	5	1.8
Renal disease	4	1.5
Liver disease	4	1.5
Hypothyroid	4	1.5
Allergy	3	1.1
Anemia	1	0.4
Diabetes	1	0.4
Migraine	1	0.4
Sinusitis	1	0.4
Favism	1	0.4
Malignancy	0	0
*Symptom*		
Asymptomatic	97	35.5
Symptomatic	176	64.5
Myalgia	81	46
Cough	80	45.5
Sore throat	68	38.6
Dyspnea	62	35.2
Headache	60	34.1
Fever	51	29
Chest pain	48	27.3
Severe weakness	46	26.1
Chills	37	21
Diarrhea	32	18.2
Anosmia	30	17
Rhinorrhea	28	15.9
Nasal congestion	28	15.9
Sweating	25	14.2
Conjunctival congestion	18	10.2
Palpitation	17	9.7
Vomiting	15	8.5
Others	4	2.3

Among the patients of our study, only 15 (5.5%) had hospital admission which the duration of hospital stays ranged from 0.5 to 8 days (mean: 3.63; SD = 2.51). Also, 53 (19.4%) had pulmonary involvement in their CT scans. It is worth mentioning that there were no ICU admitted cases among the patients in our study.

Among the patients in our study, 28 (10.3%) cases reported infected cases of COVID-19 among their family or friends after the confirmation of their infection (12 parents, nine spouses, eight children, and one friend). Furthermore, 200 (73.3%) reported a history of contact with a suspicious case in the work area, while 33 (12.1%) reported negative and 40 (14.7%) reported no knowledge of contact with infected cases.

Based on the obtained information regarding maximum applied safety precautions, considering masks, 4 (1.5%) didn’t use masks, 151 (55.3%) used surgical and standard masks, and 118 (43.22%) used N95 and FFP2 masks. Also, among the patients, 51 (18.7%) reported that they didn't use gloves in the workplace, 180 (65.9%) didn’t use protective goggles, and 159 (58.2%) didn’t use facial shields. Also, only 115 (42.1%) used gowns, 71 (26%) used special clothing and 61 (22.3%) used shoe covers in the work environment.

Post recovery symptoms of the patients included 34 (12.5%) cough, 25 (9.2%) dyspnea, 14 (5.1%) cough and dyspnea, 1 (0.4%) dyspnea and renal failure.

Based on the history of infected patients, 39 (14.3%) had no opinion, while from the remaining 234, 132 (62.3%) thought that their cause of infection to be close contact with patients, 54 (25.5%) infected and contaminated work environment, 26 (10.8%) both contact with patients and work environment, 17 (7.9%) disregard of safety precautions in contaminated workspace or contact with patients, 3 (1.4%) inadequate and unsatisfactory testing, 2 (0.9%) fatigue at work.

**Fig. 2:**
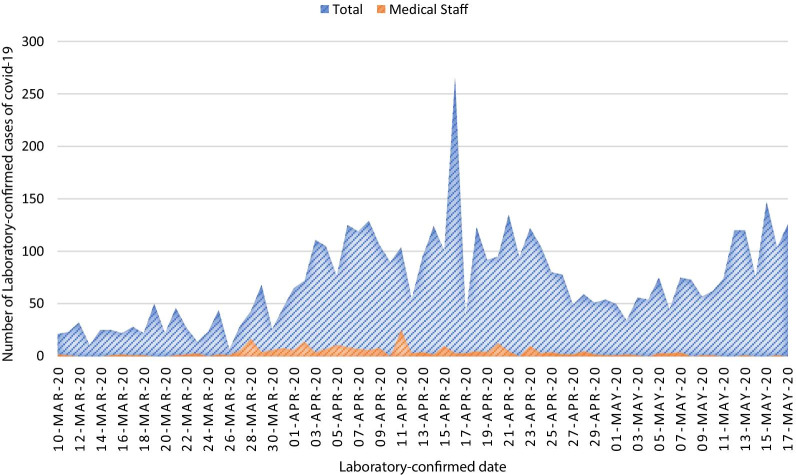
Incidence of COVID-19 infection among the general population and medical staff in Fars, Iran from 10 March to 17 May, 2020

Figure 2 demonstrates the number of infected cases with COVID-19 during the period of our study, as shown, the highest number of cases was during April.

## Discussion

The present study provides the first insight into the infection status of HCWs in Iran during the COVID-19 pandemic along with the need for proper equipment use and education of these individuals. Our report demonstrated an incidence of 273 individuals (5.62%) amongst HCW which can be assumed with a great magnitude that they acquired their disease based on occupational-related.

Based on our results the highest infection rate was among nurses (51.3%); However, the highest CIR was among physicians (3.2%). Our results are consistent with studies regarding HCW infection in Wuhan, China demonstrating a rate of 52% among nurses. This finding may be because generally, nurses have more patient contact time compared to other HCW occupations such as physicians. Focusing on educating and providing appropriate safety measures in these groups which are higher at risk is essential for their physical and mental health [10, 11].

Our study demonstrated the highest CIR to be among physicians (3.2%) which was higher than the reported CIR among physicians in China (1.92%) [12]. Our data also demonstrated that 1.5% of HCWs didn’t use masks, 18.7% didn’t use gloves, 65.9% didn’t use protective goggles and 58.2% didn’t use face shields. This data is supported by a previous study regarding the knowledge, attitude, and practice towards COVID-19 in Iran, which demonstrated that personnel with healthcare-related occupations, although with higher knowledge, had significantly lower practice towards the diseases [13]. This imposes a need for better implantation of protective measures by HCWs for decreasing infection rates in these groups.

In the early phase of the COVID-19 outbreak, the number of HCWs and personal protective equipment (PPE) was both insufficient, and the continuous working hours of HCWs were relatively longer. Therefore, the HCWs were exhausted physically and mentally. In this situation, decreased immunity and an increased chance of infection could occur in HCWs. Therefore, it is recommended that HCWs at the frontline receive sufficient rest time to ensure adequate sleep, avoid overwork and consume a nutritious diet and supplements to ensure adequate nutrition to increase the body immunity and reduce the likelihood of infection [12].

The government and the healthcare system have implanted various methods of dealing with the disease. The health ministry of Iran has dedicated several hospitals as centers for solitary COVID-19 patients, resulting in the isolation of these patients and decreasing contact, and periodic screening policy implemented in hospitals to provide early detection of the disease amongst HCWs; However there is still a high rate of infection in units such as the emergency ward (30.6%), where contact between first-hand, undiagnosed patients and HCWs would occur, and consequently, lack of awareness or diligence or vigilance of infection control, and proper safety precautions in HCWs would result in viral transmission. Among other containment methods, one can name Singapore's Ministry of Health to manage and treat all COVID-19 cases within hospitals, along with employing rapid contact tracing to detect, isolate and monitor all associates with noteworthy exposure to the index cases [14]. It should be mentioned that there was a lack of sufficient reserves and resources of hospital protective equipment for a pandemic of such magnitude. Countless medical staff are not effectively equipped and get infected by unwitting contact with carriers or COVID-19 patients. Furthermore, protective equipment, including N95 masks, protective clothing, and goggles are prioritized to first‐line HCWs in high contact centers, while other staff and centers often utmost have only surgical masks, which can result in the lower rates of infection in HCWs directly facing COVID-19 patients compared to HCWs who are less exposed.

One of the most significant findings of our study is that a considerable number of infected HCWs were asymptomatic (35.5%) and the most frequent symptoms were myalgia (46%) and cough (45.5%). Other studies have also reported a rate of 78% regarding asymptomatic COVID-19 patients [15]. This is important due to asymptomatic carriers can result in the person-to-person transmission of the disease and should be considered a source of COVID-19 infection [16]. The disease might also present with non-specific symptoms such as myalgia, which has also been reported as the most frequent (66%) syndrome in Fars province for COVID-19 in previous studies [17], making it hard to distinguish. In this regard, our data shows that 28 cases of individuals with close contact with HCWs were reported positive subsequently after the infection of the index case. The fact that detecting these individuals is challenging since they don’t present with any signs or symptoms emphasizes the need for preventing the spread of the disease compared to detecting and treating the infection to control and end the pandemic.

It is worth mentioning that among the patients of our study there was only 5.5% hospital admission with no reports of severe cases of mortality due to COVID-19, which were considerably lower than other studies. Chu et al. reported 54 hospital staff infections which included 40 severe cases and 3 critical cases (79% in total) [18], and Li et al. [19] reported 4% of the 370 hospitalized patients were severe or critical cases from Wuhan Hospital. These variations may be due to that the majority of patients in our study were under 45 years (mean 35) and 80% had no underlying diseases which makes the virus less likely to progress to a severe form in these individuals. Also, both of the mentioned studies were carried out in the initial early phase of the pandemic that viral factors in terms of its virulence and less adaptation to the human at their initial emergence might also attribute the clinical severity of COVID-19; while our study was carried out from 10th March 2020 to 17th May 2020 that was in the later phase of the pandemic compared to the mentioned studies [17, 18], in which the virus might have been further emerged to adapt in human and enhanced viral fitness, causing less severe symptoms and even more asymptomatic infection during the evolution and in the later phase of the pandemic.

It should be noted our province was affected less than other large provinces in the early phase of the pandemic and this degree of HCW involvement may change with subsequent disease surge. Healthcare infection varies based on the geographical location, magnitude, involving centers of the study, and also the method of disease confirmation. For instance, Peng et al. [20] in a single-center study in Wuhan, China reported 40 medical staff out of 138 patients (29%) while another retrospective analysis of 1099 confirmed patients with COVID‐19 in 552 hospitals from 31 provinces in China found that the proportion of health professionals was 2.09% [21]. Among other causes variations, one can name the method of including patients in the study, which in our study focused on positive RT-PCR for SARS‐CoV‐2, while in other studies, such as Chu et al. [18], reported a rate of 57 cases during 5 weeks using clinical factors according to World Health Organization interim guidance [21] The method of detection (either clinical or molecular) and which should be interpreted as the authentic number of infection rate is still a matter of debate [22]. For the purpose of this study, we only included molecular confirmed SARS-CoV2 PCR positive patients, however, although high specificity, based on the chance of false-negative the actual number of cases might much as well be considerably higher. However, it cannot be denied that HCW are amongst the highest groups at risk of infection with COVID-19, which necessitates prompt decisions and action-taking for plummeting infection rates in these individuals to continuously safeguard patient care and ensure they do not transmit the virus.

## Conclusion

With the ongoing COVID-19 outbreak and the increasing number of infected cases, ensuring the safety of HCWs is essential to end the pandemic. Therefore, investigation of the burden of COVID-19 among HCWs is vital in decreasing the spread of the virus, by increasing public awareness, and also offering useful recommendations for government agencies such as isolating infected cases in specific well-equipped hospitals. Since the majority of cases were asymptomatic, expanding protective measures for HCWs is essential to decrease infection rates among family members and colleagues.

## Supplementary Information

**Additional file 1**. Healthcare worker COVID-19 screening data collection form.

**Additional file 2**. Frequency of tests and positive cases among healthcare workers in Fars, Southern Iran.

## Data Availability

All data generated or analyzed during this study are included in this manuscript. Please write to the corresponding author for further information.

## Abbreviations

COVID-19: The novel coronavirus 2019
HCW: Health care worker
CIR: Case infection rate
PCR: Polymerase chain reaction
SARS: Severe acute respiratory syndrome
SARS-CoV-2: Severe acute respiratory syndrome coronavirus 2
ICU: Intensive care unit
WHO: World Health Organization
RT-PCR: Real-time reverse transcriptase-polymerase chain reaction
