# Supplementary material for: COVID-19 infection among healthcare workers: a cross-sectional study in southwest Iran
Source: Virol J. 2021 Mar 17;18:58. doi: 10.1186/s12985-021-01532-0 (PMC7968574; doi:10.1186/s12985-021-01532-0)
Supplement: Supplementary file 1 — Additional file 1. Healthcare worker COVID-19 screening data collection form. [file 12985_2021_1532_MOESM1_ESM.docx]

**Healthcare worker COVID-19 screening data collection form**

1. Age:

| Male |  | Female |  |
| --- | --- | --- | --- |

2. Gender:

3. Occupation

| General Practitioner |  | Specialist doctor  (Please mention field) |  |
| --- | --- | --- | --- |
| Nurse |  | Midwife |  |
| Staff |  | Resident |  |
| Medical Student |  | Nursing student |  |
| Fellowship (Please mention field) |  | Radiology Technician |  |
| Physiotherapist |  | Services/ Janitor |  |
| Laboratory personnel |  | Others (Please name) |  |

4. Work experience: _____ years

5. Work location (checking multiple options is permitted)

| City |  | Hospital |  |
| --- | --- | --- | --- |
| Clinic |  | Wards |  |
| Emergency department |  | Operation room |  |
| Radiology Center |  | Laboratory |  |
| Reception |  | Security |  |
| Others (Please mention) |  | | |

6. Do you have a history of underlying disease? Please mention its type (for example, in heart disease, if heart failure or arrhythmia or ....) (checking multiple options is permitted)

| Heart disease |  | Respiratory disease |  | Renal disease |  |
| --- | --- | --- | --- | --- | --- |
| Liver disease |  | Gastrointestinal tract disease |  | Cancer |  |
| Immunodeficiency |  | Corticosteroid use |  | Chemotherapy |  |
| Other (Please name) |  | | | | |

7. What were your symptoms at the time of diagnosis with COVID-19? (checking multiple options is permitted)

| Fever |  | Decrease level of consciousness |  |
| --- | --- | --- | --- |
| Chills |  | Cough |  |
| Dyspnea |  | Loss of sense of smell/taste |  |
| Headache |  | General weakness/ Malaise |  |
| Body pain |  | Sore throat |  |
| Diarrhea |  | Sweating |  |
| Vomiting |  | Redness of eyes |  |
| Palpitation |  | Chest pain |  |
| Nasal congestion or runny nose |  | No symptoms |  |
| Others (Please describe) |  | | |

8. How many times have you taken the PCR test for COVID-19? What date? Positive or negative? (If the test answer is unclear for any reason, please check others)

| Row | Date | Positive (+) | Negative (-) | Other |
| --- | --- | --- | --- | --- |
| 1 |  |  |  |  |
| 2 |  |  |  |  |
| 3 |  |  |  |  |
| 4 |  |  |  |  |
| 5 |  |  |  |  |

9. Have you been hospitalized during your COVID-19 illness? On what date and how many days?

| Date |  | Duration |  |
| --- | --- | --- | --- |

10. Have you been admitted to the ICU during your COVID-19 illness? On what date and how many days?

| Date |  | Duration |  |
| --- | --- | --- | --- |

11. Have you received mechanical ventilation? How many days?

| Date |  | Duration |  |
| --- | --- | --- | --- |

12. Has anyone else close to you contracted the COVID-19 before your illness? (checking multiple options is permitted)

| Spouse |  | Children |  | Father/Mother |  | Friends |  | Other |  |
| --- | --- | --- | --- | --- | --- | --- | --- | --- | --- |

13. Has anyone else in close to you contracted the COVID-19 after your illness? (checking multiple options is permitted)

| Spouse |  | Children |  | Father/Mother |  | Friends |  | Other |  |
| --- | --- | --- | --- | --- | --- | --- | --- | --- | --- |

14. Have you been in contact with a suspicious COVID-19 case outside of the hospital before your illness? Who?

| Yes |  | No |  | Don’t know |  |
| --- | --- | --- | --- | --- | --- |
| Relation: | | | | | |
| First class relative |  | Second class relative |  | Friends |  |

15. Have you been in contact with a suspicious COVID-19 case in your work area?

| Yes |  | No |  | Don’t know |  |
| --- | --- | --- | --- | --- | --- |

16. What kind of personal protection equipment did you use before getting infected with the COVID-19? (checking multiple options is permitted)

| Normal/Surgical Mask |  | N95/FFP2 Mask |  |
| --- | --- | --- | --- |
| Goggles |  | Face Shield |  |
| Normal/Surgical Gloves |  | Gowns |  |
| Shoe cover |  | Special overall clothing |  |
