# Supplementary material for: COVID-19 infection among healthcare workers: a cross-sectional study in southwest Iran
Source: Virol J. 2021 Mar 17;18:58. doi: 10.1186/s12985-021-01532-0 (PMC7968574; doi:10.1186/s12985-021-01532-0)
Supplement: Supplementary file 2 — Additional file 2. Frequency of tests and positive cases among healthcare workers in Fars, Southern Iran. [file 12985_2021_1532_MOESM2_ESM.docx]

**Supplementary Table 2.** Frequency of tests and positive cases among healthcare workers in Fars, Southern Iran

| Hospital Name | City | Total *n=10707* | Doctor *n=842* | Nurse *n=6183* | Paraclinical *n=1165* | Office *n=2517* | Positive Cases *n=273* |
| --- | --- | --- | --- | --- | --- | --- | --- |
| Imam Khomeini | Abadeh | 130 | 16 | 78 | 9 | 27 | 0 |
| Arsenjan | Arsenjan | 127 | 10 | 73 | 15 | 29 | 2 |
| Valiasr Hospital | Bavanat | 115 | 9 | 64 | 31 | 11 | 3 |
| Imam Khomeini | Estahban | 74 | 6 | 59 | 0 | 9 | 9 |
| Qaem hospital | Firuzabad | 195 | 7 | 118 | 27 | 43 | 0 |
| Imam Mohammad Bagher | Ghirokarzin | 127 | 5 | 83 | 17 | 22 | 8 |
| Fateme Zahra Hospital | Kavar | 13 | 2 | 9 | 1 | 1 | 3 |
| Kazeroon Hospital | Kazeroon | 217 | 15 | 156 | 9 | 37 | 6 |
| JavadAlaeme Hospital | Kharameh | 76 | 3 | 38 | 13 | 22 | 0 |
| Emam Sajjad Hospital | Khesht | 13 | 1 | 5 | 2 | 5 | 16 |
| Khorrambid | Khorrambid | 18 | 0 | 12 | 0 | 6 | 0 |
| Lamerd Hospital | Lamerd | 120 | 6 | 100 | 12 | 2 | 43 |
| Noorabad | Mamasani | 110 | 3 | 90 | 5 | 12 | 0 |
| Motahari Hospital | Marvdasht | 655 | 25 | 352 | 122 | 156 | 25 |
| Shohada Hospital | Neyriz | 86 | 6 | 65 | 4 | 11 | 4 |
| Pasargard | Pasargard | 95 | 7 | 47 | 8 | 33 | 11 |
| Qaemyeh Hospital | Qaemyeh | 64 | 1 | 20 | 10 | 33 | 0 |
| Sarvestan Hospital | Sarvestan | 28 | 3 | 15 | 1 | 9 | 1 |
| Sepidan Hospital | Sepidan | 136 | 11 | 64 | 42 | 19 | 4 |
| Mirhosseini Hospital | Shiraz | 244 | 12 | 120 | 5 | 107 | 4 |
| Ebn Sina Hospital | Shiraz | 115 | 16 | 63 | 11 | 25 | 0 |
| Zeinabiyyeh Hospital | Shiraz | 305 | 35 | 195 | 14 | 61 | 3 |
| Abu-Ali Sina Hospital | Shiraz | 439 | 33 | 251 | 59 | 96 | 3 |
| Ordibehesht Hospital | Shiraz | 68 | 5 | 25 | 3 | 35 | 2 |
| Shahid Dastgheib Hospital | Shiraz | 198 | 21 | 73 | 14 | 90 | 5 |
| Emtyaz Hospital (Trauma Center) | Shiraz | 329 | 23 | 219 | 22 | 65 | 2 |
| Shahriar Hospital | Shiraz | 229 | 26 | 98 | 12 | 93 | 0 |
| Moslemin Hospital | Shiraz | 226 | 4 | 80 | 97 | 45 | 6 |
| Namazi Hospital | Shiraz | 1454 | 105 | 1092 | 125 | 132 | 22 |
| Professor Moharari Psychiatric Hospital | Shiraz | 313 | 17 | 164 | 6 | 126 | 20 |
| Hafez Hospital | Shiraz | 255 | 32 | 170 | 10 | 43 | 4 |
| Shiraz Central Hospital | Shiraz | 350 | 34 | 178 | 38 | 100 | 7 |
| Motahari Clinic | Shiraz | 236 | 15 | 85 | 0 | 136 | 4 |
| Faqihi Hospital | Shiraz | 1092 | 171 | 595 | 116 | 210 | 16 |
| Artesh Hospital | Shiraz | 70 | 10 | 30 | 6 | 24 | 1 |
| Khalili Hospital | Shiraz | 150 | 40 | 44 | 41 | 25 | 1 |
| Kowsar Hospital | Shiraz | 400 | 13 | 240 | 28 | 119 | 0 |
| Pars Hospital | Shiraz | 134 | 2 | 65 | 2 | 65 | 0 |
| Shoushtari Hospital | Shiraz | 258 | 20 | 119 | 25 | 94 | 0 |
| Chamran | Shiraz | 380 | 29 | 221 | 31 | 99 | 13 |
| Doran Hospital | Shiraz | 230 | 21 | 103 | 54 | 52 | 7 |
| Al-Zahra Heart Hospital | Shiraz | 740 | 10 | 464 | 104 | 162 | 15 |
| Imam Hasan Askari | Zarghan | 55 | 8 | 27 | 11 | 9 | 3 |
| Zarrin Dasht Hospital | Zarrin Dasht | 38 | 4 | 14 | 3 | 17 | 0 |
